# Genome-Wide Identification and Evolutionary Analysis of the SBP-Box Gene Family in Castor Bean

**DOI:** 10.1371/journal.pone.0086688

**Published:** 2014-01-22

**Authors:** Shu-Dong Zhang, Li-Zhen Ling

**Affiliations:** 1 Key Laboratory of Biodiversity and Biogeography, Kunming Institute of Botany, the Chinese Academy of Sciences, Kunming, China; 2 Plant Germplasm and Genomics Center, Germplasm Bank of Wild Species, Kunming Institute of Botany, the Chinese Academy of Sciences, Kunming, China; University of South Florida College of Medicine, United States of America

## Abstract

Genes in the SQUAMOSA promoter-binding-protein (SBP-box) gene family encode transcriptional regulators and perform a variety of regulatory functions that involved in the developmental and physiological processes of plants. In this study, a comprehensive computational analysis identified 15 candidates of the SBP-box gene family in the castor bean (*Ricinus communis*). The phylogenetic and domain analysis indicated that these genes were divided into two groups (group I and II). The group II was a big branch and was further classified into three subgroups (subgroup II-1 to 3) based on the phylogeny, gene structures and conserved motifs. It was observed that the genes of subgroup II-1 had distinct evolutionary features from those of the other two subgroups, however, were more similar to those of group I. Therefore, we inferred that group I and subgroup II-1 might retain ancient signals, whereas the subgroup II-2 and 3 exhibited the divergence during evolutionary process. Estimation of evolutionary parameters (d_N_ and d_N_/d_S_) further supported our hypothesis. At first, the group I was more constrained by strong purifying selection and evolved slowly with a lower substitution rate than group II. As regards the three subgroups, subgroup II-1 had the lowest rate of substitution and was under strong purifying selection. By contrast, subgroups II-2 and 3 evolved more rapidly and experienced less purifying selection. These results indicated that the different evolutionary rates and selection strength caused the different evolutionary patterns of the members of SBP-box genes in castor bean. Taken together, these results provide better insights into understanding evolutionary divergence of the members of SBP-box gene family in castor bean and provide a guide for future functional diverse analyses of this gene family.

## Introduction

Precise and coordinate gene expression is essential for organism growth and development. Regulation of gene transcription is the primary one among the many mechanisms that operate to control gene expression. Transcriptional control relies on transcription factors (TFs), which are usually defined as proteins that show sequence-specific DNA binding and are capable of activating and/or repressing transcription. Most known transcription factors can be grouped into families according to their DNA binding domain [1]. These domains are evolutionarily conservative within their respective families, and enable definition of over thirty such transcription factor families in the model plant *Arabidopsis thaliana* [2].

*SQUAMOSA* (*SQUA*) promoter-binding-like (SPL) genes represent one such family of plant-specific transcription factors [3]. Each member of this family contains a highly conserved DNA-binding domain (*SQUAMOSA* promoter-binding-protein (SBP) domain) with two separate zinc-binding sites (one zinc finger is C3H or C4, and the other is C2HC [4]). These SPL genes are known to have important functions in the transcriptional regulation of a variety of biological processes related to growth and development, and in controlling various responses to environmental stimuli [5]. However, a precise annotation of SPL gene is the first step to fully understanding their roles. After the release of whole genome sequences or large sets of expressed sequence tags for plants or algae, researchers typically identify and describe all the associated SPL genes [6]–[10]. To date, all members of the SBP-box gene family from 15 plant species have been deposited in the Plant Transcription Factor database (PlnTFDB) [11]. With the implication of high throughput sequencing technology, more and more plant genomes — such as apple (*Malus domestica*) [12], cucumber (*Cucumis sativus*) [13], soybean (*Glycine max*) [14] and watermelon (*Citrullus lanatus*) [15] — have been sequenced recently. A great deal of experimental work will be required to determine the specific biological function of each of the SPL genes in these plant species. Two previous studies reported that the SBP-box gene family consists of two groups (I and II), based on phylogenetic analyses [9], [16]. Group II includes many distinct sets of genes that are closely related to each other, so it was further divided into seven subgroups (IIa–IIg). These data indicated that SPL genes generate functional diversity during evolution, because genes within a subgroup are likely to share similar functions. Structural relationships among SPL proteins, along with identification of putative motifs, provide additional evidence for these diversities.

The draft genome sequence has been published for the castor bean (*Ricinus communis*) an important oil-producing member of the Spurge family (Euphorbiaceae) that is the only species its genus and has no immediate relatives [17]. However, information about the homologous SPL genes of this plant has not yet been reported. Important questions about the diversity of these genes remain to be addressed. Which SPL genes are specific to the castor bean? What is the evolutionary relationship between SPL genes in the castor bean and other plants? What does the unique evolutionary history of each castor bean SPL gene reveal about genetic functional diversity?

In this study, we addressed these questions to establish a complete picture of the SBP-box gene family in the castor bean. A total of 15 SPL genes was identified using BLAST to search genome and protein sequence databases. An overview of this gene family is presented, including the gene structures, phylogeny, and conserved motifs found in the castor bean. Results of a comparative analysis of the substitution rates between groups and subgroups are also shown. Finally, we discuss the relationship between the substitution rate and the expression level and protein length of SPL genes.

## Results and Discussion

### Identification of SBP-box genes in castor bean

**Figure 1:**
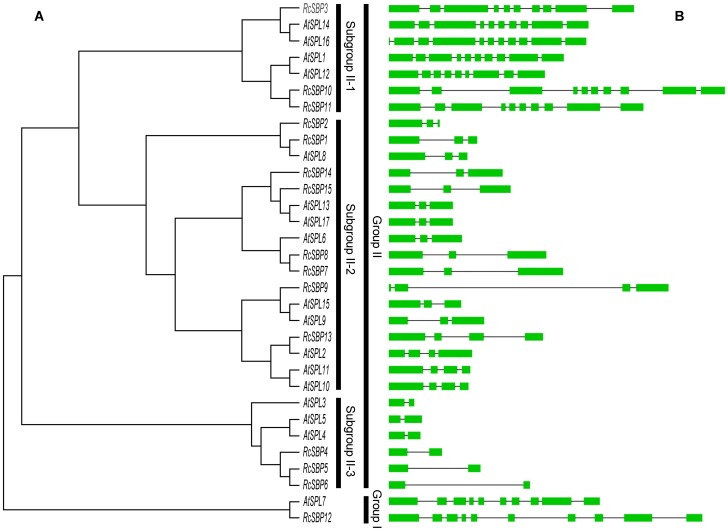
Neighbor-Joining phylogenetic tree of the SBP domains of *Arabidopsis* and castor bean (A) and the corresponding gene structure analyses (B). Green boxes indicate the exon regions and lines indicate introns. The length of the boxes and lines are scaled based on the length of genes.

To identify the SBP-box genes present in the castor bean, we used multiple BLAST algorithms to compare against the genome and protein data sets, using the representative SBP domains of *Arabidopsis* for each subgroup as our query (see Materials and Methods). Twenty-seven genes were initially identified as candidates that possess possibly the SBP domain. Following the removal of redundant sequences and SMART [18] analysis, we identified at least 15 putative SPL genes in the castor bean genome; in the absence of an existing nomenclature for them, we used the gene model and locus as identifiers (Table 1). Previous annotations of SBP-box genes in rice (*Oryza sativa*) [10] and the common grape (*Vitis vinifera*) [19] were based on chromosomal order. Such information about chromosome location of SPL genes in the castor bean was unavailable, so we proposed an alternative numbering system for the SBP-box genes based on the *Arabidopsis* homolog. A similar method has been used to name the SPL genes in the tomato plant (*Solanum lycopersicum*) [20]. Based on that approach and our phylogenetic analysis (Fig. 1A), we assigned a provisional, generic name as a unique identifier for each SPL gene (*RcSBP1* to *RcSBP15*) in this study.

**Table 1:** Summary of information on the SPL genes identified in castor bean.

Gene_name	Scaffold	Locus_id	Gene_model	Group/Subgroup	Length^a^
***RcSBP1***	30174	30174.t000200	30174.m008803	II-2	302
***RcSBP2***	30174	30174.t000202	30174.m008805	II-2	262
***RcSBP3***	30170	30170.t000657	30170.m014245	II-1	1073
***RcSBP4***	28211	28211.t000006	28211.m000132	II-3	198
***RcSBP5***	29983	29983.t000057	29983.m003158	II-3	198
***RcSBP6***	30190	30190.t000565	30190.m011329	II-3	141
***RcSBP7***	30138	30138.t000116	30138.m003942	II-2	557
***RcSBP8***	30174	30174.t000479	30174.m009082	II-2	512
***RcSBP9***	29269	29269.t000012	29269.m000250	II-2	349
***RcSBP10***	29889	29889.t000154	29889.m003388	II-1	1026
***RcSBP11***	30076	30076.t000138	30076.m004574	II-1	1012
***RcSBP12***	29726	29726.t000071	29726.m003959	I	795
***RcSBP13***	29929	29929.t000144	29929.m004641	II-2	483
***RcSBP14***	29929	29929.t000130	29929.m004627	II-2	404
***RcSBP15***	30147	30147.t000749	30147.m014478	II-2	382

a The length indicates the protein length of each gene.

To examine the typical domains of all putative SPL proteins, multiple alignment analyses were performed. The alignments indicated that all SPL proteins contained the complete SBP domain; each of them contained approximately 79 amino acid residues and a sequence-specific DNA-binding domain (DBD) (Fig. S1). This DBD contained two zinc-binding sites and a bipartite nuclear localization signal (NLS) in the N-terminal [21] (Fig. S1). The alignments also indicated that these SBP domains were highly conserved. Amino acid sequences for the DBD of all proteins were identical, except for one residue in the first Zn finger-like structure. Within 14 proteins, the first structure was assembled as Cys-Cys-His-Cys (Cys2HisCys), whereas the Cys residue replaced the His within one remaining protein (*RcSBP12*). In our previous study [16], the SPL genes with the distinct SBP domain were defined two groups (group I and group II). The genes in group I contained four Cys residue, but those belong to group II had a Cys3His motif in the first Zn finger-like structure. Similarly, the proteins in this study were also assigned into two groups: *RcSBP12* was classified as group I and the remaining 14 proteins were assigned to group II (see Fig. 1A).

### Phylogenetic relationships of SBP-box genes in castor bean and *Arabidopsis*

To describe the SPL genes of castor bean and determine their evolutionary relationship with *Arabidopsis* genes, we generated a phylogenetic tree based on the highly conserved SBP domains. Castor bean SPL genes were not clustered together in a species-specific manner (Figure 1A), even though it is the only species within *Ricinus*. In contrast, they were scattered throughout the phylogenetic tree, much like *Arabidopsis* SPL genes. This result suggested that the SPL genes of castor bean originated prior to the speciation of castor bean and *Arabidopsis*. We further estimated timing of the origin of the castor bean ones by adding the SPL genes of a species of moss into the phylogenetic analysis. The resulting phylogenetic tree revealed that some castor bean SPL genes clustered together with those from moss (data not shown), suggesting that the castor bean SPL genes might have originated up to 400 million years ago. In addition, we observed that each phylogenetic branch contained at least one *Arabidopsis* and castor bean SBP-box protein. This suggests that SPL genes from the two species have similar functions. To better understand their evolutionary relationships and functions, we analyzed a synteny map to compare the two genomes. The resulting, putative ortholog pairs were: *RcSBP12-AtSPL7*; *RcSBP10/11-AtSPL1*; *RcSBP13-AtSPL11*; *RcSBP14-AtSPL13*; *RcSBP5-AtSPL4*; *RcSBP7*/*8-AtSPL6*; *RcSBP15-AtSPL17*; *RcSBP3-AtSPL14* (Figure S2). *Arabidopsis* is an important model species, so the functions of most members of its SBP-box gene family have been well-characterized. For example, *Arabidopsis* SPL genes are reportedly involved in divergent biological processes, including leaf development, sporogenesis, phase change, and response to copper homeostasis [22], [23]. We inferred that castor bean SPL genes might be as functionally diverse as those of *Arabidopsis*. Taken together, our results suggest that the castor bean SPL genes have a deep evolutionary origin and might control a considerable variation of biological functions.

The phylogenetic tree indicated that one of the fifteen genes (*RcSBP12*) was classified as part of group I along with *AtSPL7*, while the remainder constituted group II (Fig. 1A). This classification was consistent with the domain analysis described above. Group II was a big branch and further resolved into three subgroups, namely, subgroup II-1 to 3 (Fig. 1A), rather than the seven major subgroups that have been described in previous studies (subgroup II a-g) [16]. There was a clear parallel relationship between branches and the gene structures (Figure 1B). In subgroup II-1, all the SPL genes shared a conserved splicing pattern of ten exons and nine introns, while subgroup II-2 were comprised of three/four exons and two/three introns, subgroup II-3 with two core exons and one intron, respectively. The apparent correlation between gene structures and the classes of SPL genes was probably due to the expansion of SPL genes in each clade during ancient and recent duplication events. An alternative possibility was that the exon/intron structure of SPL genes could have a certain level of stability during the late stages of evolution of two species. Likewise, MEME analysis revealed extensive conservation in motif architecture within the SPL genes of each subgroup. For example, twelve motifs were identified in seven genes from subgroup II-1, and these motifs occupied the same relative positions in seven protein sequences. Therefore, the motif analysis further validated our classification of castor bean SPL genes in this study. Nevertheless, we noticed that SPL genes in the branch of subgroup II-1 and group I were surprisingly similar in several respects. First, the number of exons and introns of SPL genes from these two clades was almost the same (Fig. 1B). Second, the SPL genes from subgroup II-1 and group I possessed the same motifs (4, 12 and 13) with the exception of the conserved SBP-box domain (Figure S3). Finally, the lengths of proteins in the subgroup II-1 and group I were, on average, longer than those of subgroup II-2 and 3 (Table 1 and Table S1). These distinct characters of subgroup II-1 were significantly different from those of subgroup II-2 and 3. All in all, these comparisons implied that the SPL genes of group II might be diverse after divergence from those of group I. Among three subgroups of group II, the SPL genes of subgroup II-1 were yet likely to retain ancient evolutionary signals. By contrast, the SPL genes of subgroup II-2 and 3 exhibited the distinct evolutionary characters since their origin via gene duplication.

### Diverse substitution rates for groups and subgroups

**Figure 2:**
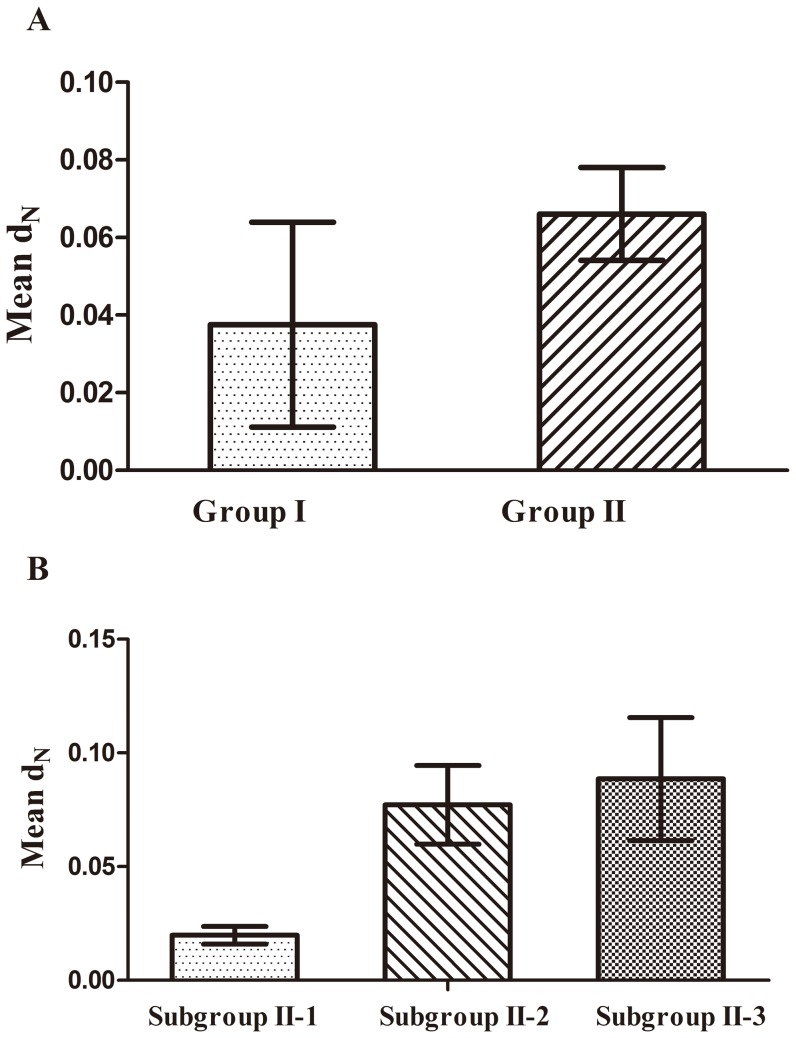
Comparison of the mean d_N_ value for two groups (A) and three subgroups (B). Error bars indicate the standard error of the mean.

We hypothesized that the genes most likely to retain ancient evolutionary signals should be in the most slowly evolving positions. To test this, we estimated synonymous (d_S_) and nonsynonymous (d_N_) substitution rates for the SPL genes of two groups and three subgroups, separately. Synonymous substitutions usually accumulate more rapidly than nonsynonymous ones, which leads to a differential saturation of d_S_ and d_N_, thus potentially biasing estimates of their values. On the other hand, the saturation of synonymous substitutions could mask the information provided by nonsynonymous ones. To avoid that problem with synonymous substitution rates, we used only nonsynonymous substitution rates. The mean d_N_ value for group I was two times lower than that for group II (Figure 2A). This indicated that the genes of group I are evolving significantly slower than those of group II at the nonsynonymous sites. The relative occurrence of nonsynonymous substitutions is used to represent the strength of selective pressure [24]. Therefore, we calculated the ratio of d_N_/d_S_ using the tree topology suggested by neighbor-joining (NJ) analysis of the domain sequences. We found that d_N_/d_S_ values were much lower than 1.0 for the two groups, suggesting that purifying selection was operating: the ratio was estimated as 0.08 for group I and 0.31 for group II. We analyzed the three subgroups in the same way. First, we found that the substitution rate was significantly slower in the genes of subgroup II-1 than in those of subgroups II-2 and 3 (Fig. 2B). However, the genes of the latter two subgroups were evolving at a similar rate. In addition, we detected the d_N_/d_S_ ratio for each subgroup. The genes of subgroup II-1 had a lower d_N_/d_S_ ratio, whereas the genes of the other two subgroups had elevated ratios (d_N_/d_S_ = 0.04 for subgroup II-1, 0.86 for subgroup II-2, and 0.13 for subgroup 3), which was consistent with results of the d_N_ analysis. This suggests that the genes of subgroup II-1 were subjected to stronger selection pressures and have evolved more slowly than those of subgroup II-2 and 3.

Our results indicate that the genes from different groups and subgroups displayed different evolutionary patterns. The genes of group I were constrained by strong purifying selection, and evolved conservatively at a slower rate. By contrast, group II exhibited more complex evolutionary patterns and was further divided into three subgroups that exhibited markedly different evolutionary diversity following divergence from group I. Among the three subgroups, only the genes of subgroup II-1 possessed similar evolutionary characteristics compared to group I. These genes also had a lower substitution rate compared to genes from the other two subgroups. These relationships indicate that the genes from group I and subgroup II-1 evolved slowly and retained ancient signals (i.e. more exons and introns). Therefore, we inferred that the SPL ancestor gene might have a complex gene structure with many exons and introns. The genes of subgroup II-2 and 3 might have suffered exon and intron loss during the evolutionary process.

When we analyzed the SPL genes of the castor bean alone, a similar trend was observed. Previous studies have examined that many plausible, functional constraints have been related to the different evolutionary patterns (in particular the evolutionary rate) [25], [26]. In our study, we first considered the expression level because it seemed to be strongest predictor of evolutionary rates. However, our results indicated that the rate of evolution of castor bean SPL genes was independent of the intensity of their expression (unpublished data). Moreover, protein structure, the length of proteins or UTRs, and other factors may also affect heterogeneous evolutionary rates [27]–[29]. We observed that the divergence rates for protein sequences of different subgroups seemed to be correlated with their protein lengths (Table 1, Table S1 and Fig. 2B). Although the lengths of the genes varied among different subgroups, they remained similar within each subgroup. Furthermore, the proteins of subgroup II-1 were apparently longer than those of the other two subgroups, but were of similar length to those from group I. Our results therefore indicate that these slowly evolving genes had longer protein sequences, while the rapidly evolving genes had shorter protein sequences. Whether protein structure and other factors also have an important influence on the divergence of evolutionary rates of the genes from different subgroups requires further investigation.

## Conclusions

These results provide strong supporting evidence for the evolutionary diversity of SPL genes in the castor bean. Our novel study provides a foundation for analyzing the functional diversification of the members in the SBP-box gene family in castor bean.

## Materials and Methods

### Database Search

To begin, all members of the *Arabidopsis* SBP-box gene family were downloaded from the PlnTFDB database [11]. One random *Arabidopsis* SPL gene for each group/subgroup defined by us [16] was then selected as the query. Subsequently, the domain sequences of these representative members were used to retrieve the castor bean genome database using the BLAST program [30]. In addition, we searched the castor bean protein database using these representative sequences, to confirm that the search had been exhaustive. Based on the thorough search, we collected all members of the castor bean SBP-box gene family from currently available genomic and protein databases (http://castorbean.jcvi.org/downloads.php) (Table 1). As a final quality check, we confirmed the presence of the SBP domain in every SPL candidate gene using SMART [18].

### Sequence Alignments and Phylogenetic Analyses

The SBP-box domain sequences from castor bean and *Arabidopsis* were first aligned using ClustalX [31], and then refined manually. Next, the neighbor-joining (NJ) method was used to construct the phylogenetic tree using the PHYLIP software suite (v.3.6) (http://evolution.genetics.washington.edu/phylip.html). The amino acid substitution matrix used was the Jones-Taylor-Thornton (JTT) model, and local support values were assessed using 1000 replicate bootstrap tests.

### Exon/Intron Structure and Motif Analysis

Exon/intron site and length data were extracted based on the genome annotation GFF files (http://castorbean.jcvi.org/downloads.php). A diagram of exon/intron structures was created using the online Gene Structure Di*SPLay* Server (GSDS, http://gsds.cbi.pku.edu.cn/). Motifs were detected using MEME (version 4.9.0) [32] with the following parameter settings: distribution of motifs  =  zero or one per sequence; maximum number of motifs to find = 15; minimum width of motif = 6; maximum width of motif = 80; minimum number of sites for each motif = 2 (that is the minimum number of sequences of subgroup/group). All other options were set at default values.

### Estimating Substitution Rates

Synonymous (d_S_) and nonsynonymous (d_N_) substitution rates per protein were estimated using the program *codeml* in PAML version 4.4 [32]. Since synonymous substitutions do not change protein products, and are therefore putatively neutral, they are thought to accumulate at an approximately constant rate. However, with increasing evolutionary age, the variation of substitution sites might eventually saturate. That is, the d_S_ estimates will be systematically lower than the real synonymous substitution levels. To avoid the problem of saturation of synonymous substitutions, we applied a threshold that is usually set around d_S_>1.5 in many studies.

## Supporting Information

Figure S1 **Multiple sequence alignments of the SBP domains of the 15 members of the SBP-box gene family in castor bean.** Black and light gray shading indicate identical and conserved amino acid residues, respectively. The Cys residue of *RcSBP 12* which was different from the corresponding one of the remaining 14 proteins in the first Zn finger-like structure was marked by the red box.
(TIF)

Figure S2 **Conserved synteny of castor bean and** ***Arabidopsis*** **SPL genes.** The scaffolds of castor bean and the chromosomes of *Arabidopsis* are depicted as horizontal gray and blue bars, respectively. Castor bean and *Arabidopsis* SPL genes are indicated by vertical black lines. The marked regions in the scaffolds of castor bean and the chromosomes of *Arabidopsis* denote syntenic regions. Note: The length of the bars is scaled based on the length of scaffolds of castor bean. However, the length of chromosomes of *Arabidopsis* is too long and denoted by “//”.
(TIF)

Figure S3 **Schematic diagram of motif architectures of every group or subgroup.** The length of the motif can be estimated using the scale at bottom.
(TIF)

Table S1 **Summary of information on** ***Arabidopsis*** **SPL genes used in this study.**
(DOC)
