# Supplementary figures and images for: Genome-Wide Identification and Evolutionary Analysis of the SBP-Box Gene Family in Castor Bean
Source: PLoS One. 2014 Jan 22;9(1):e86688. doi: 10.1371/journal.pone.0086688 (PMC3899293; doi:10.1371/journal.pone.0086688)

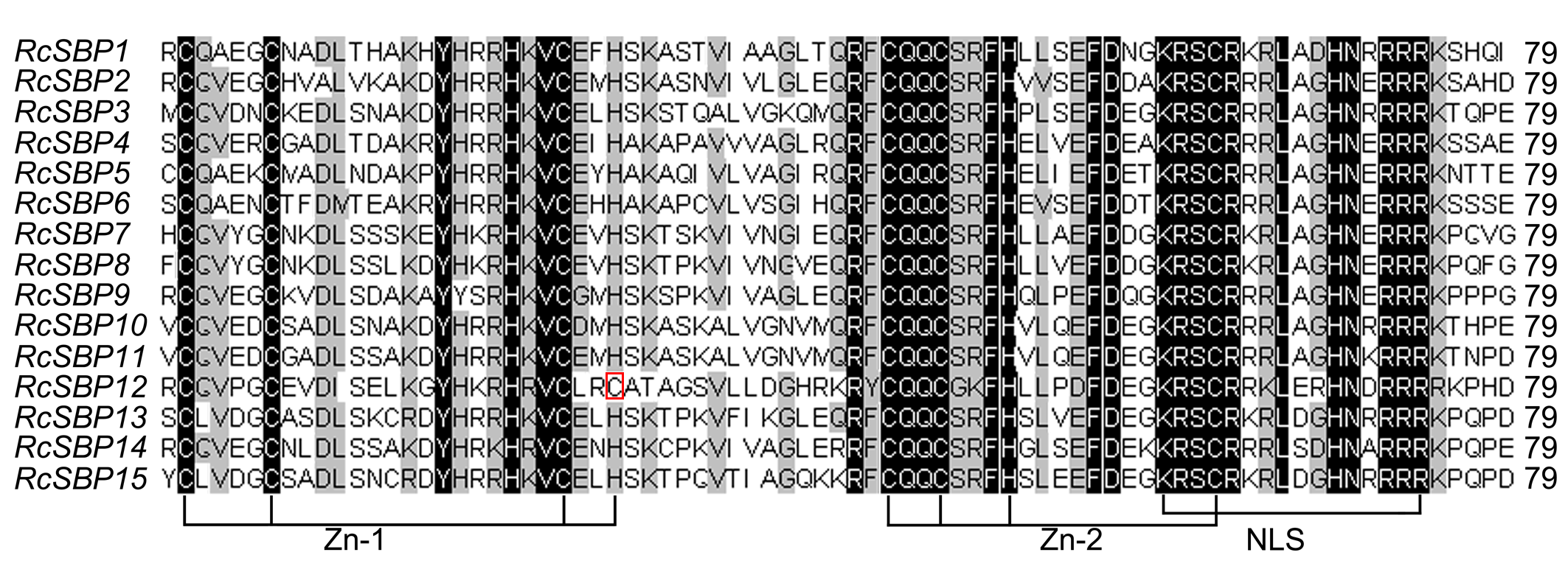

Supplement: Figure S1 — Multiple sequence alignments of the SBP domains of the 15 members of the SBP-box gene family in castor bean. Black and light gray shading indicate identical and conserved amino acid residues, respectively. The Cys residue of RcSBP 12 which was different from the corresponding one of the remaining 14 proteins in the first Zn finger-like structure was marked by the red box. (TIF) [file pone.0086688.s001.tif]

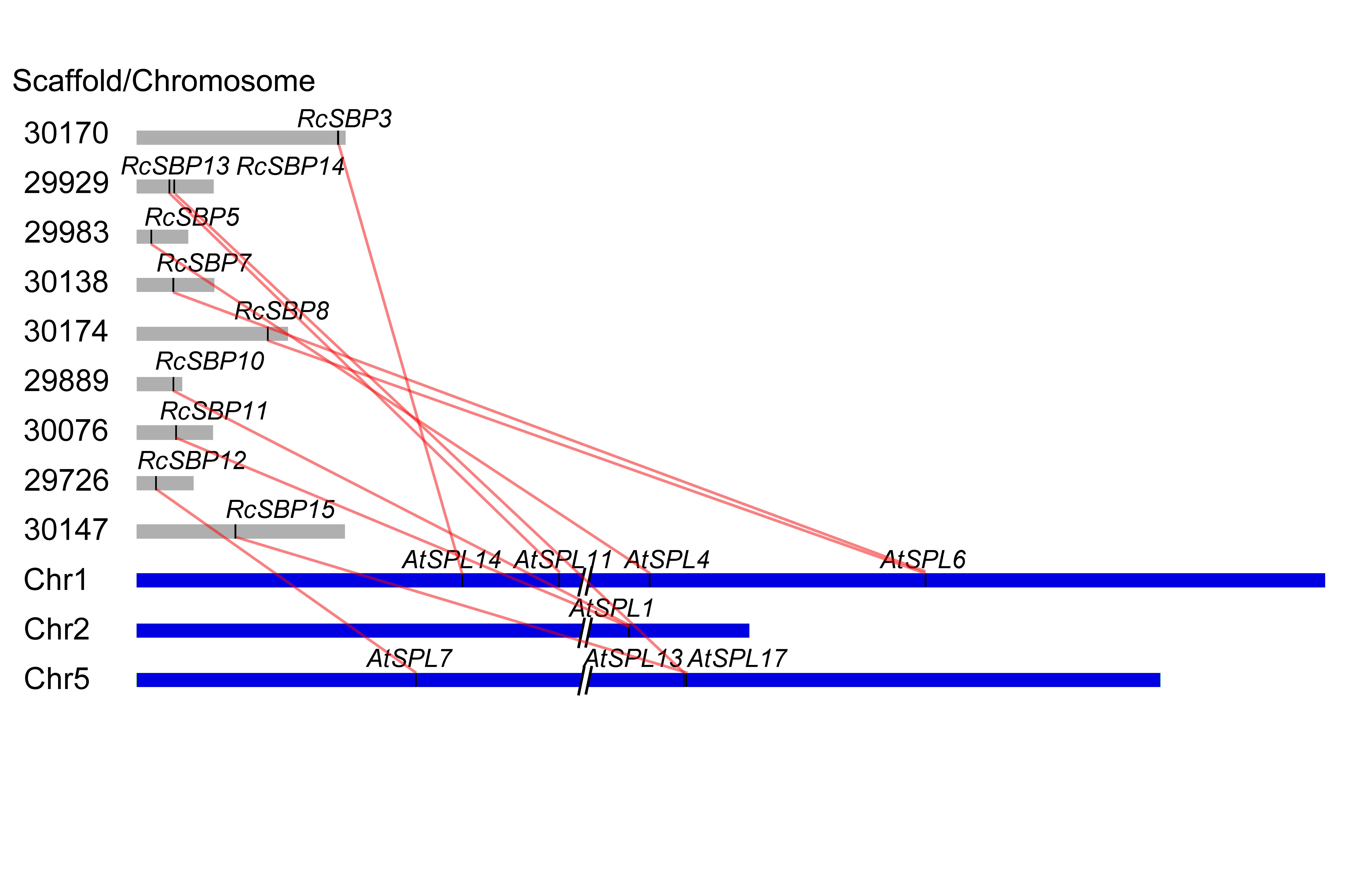

Supplement: Figure S2 — Conserved synteny of castor bean and Arabidopsis SPL genes. The scaffolds of castor bean and the chromosomes of Arabidopsis are depicted as horizontal gray and blue bars, respectively. Castor bean and Arabidopsis SPL genes are indicated by vertical black lines. The marked regions in the scaffolds of castor bean and the chromosomes of Arabidopsis denote syntenic regions. Note: The length of the bars is scaled based on the length of scaffolds of castor bean. However, the length of chromosomes of Arabidopsis is too long and denoted by “//”. (TIF) [file pone.0086688.s002.tif]

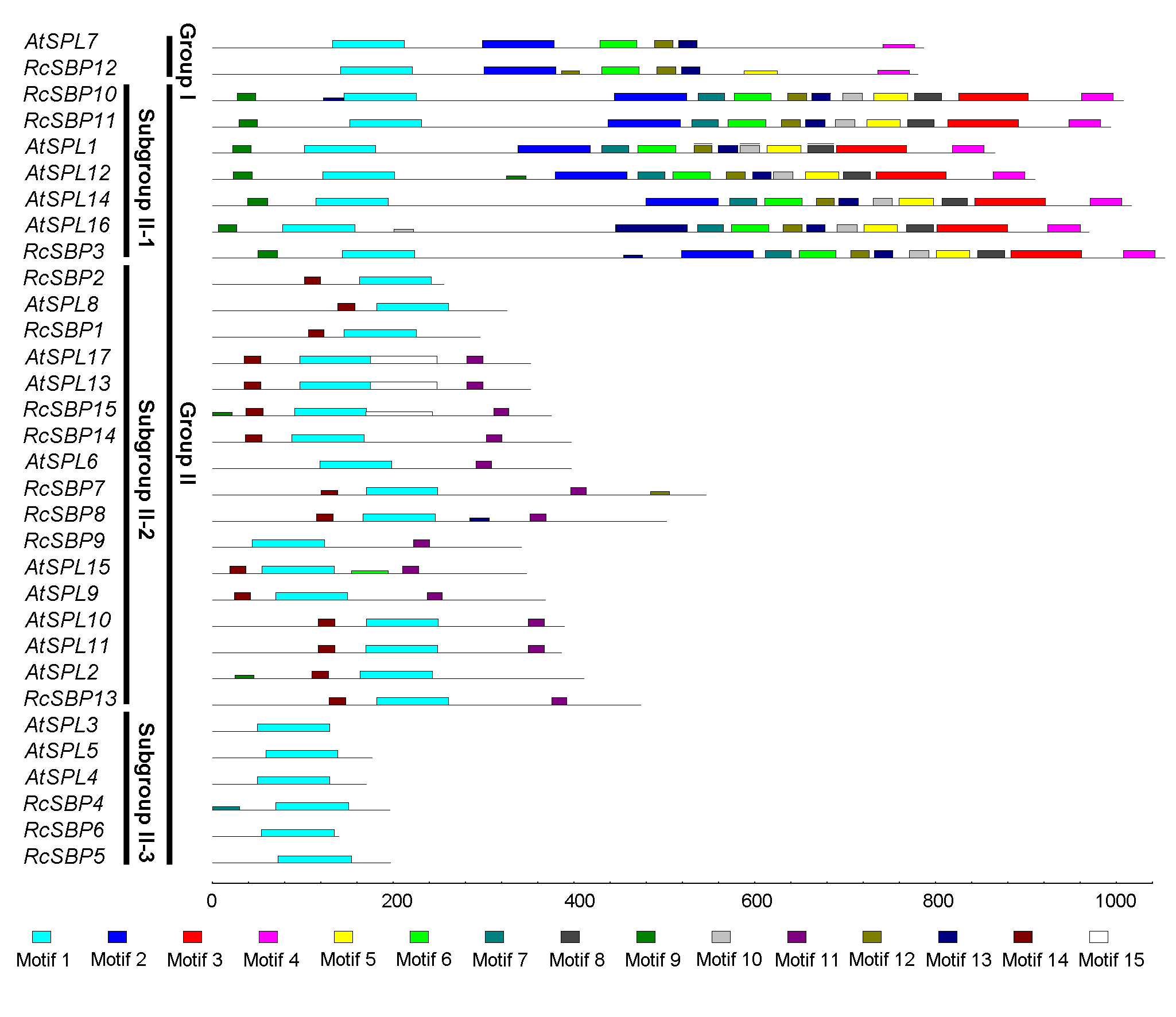

Supplement: Figure S3 — Schematic diagram of motif architectures of every group or subgroup. The length of the motif can be estimated using the scale at bottom. (TIF) [file pone.0086688.s003.tif]
