# Supplementary material for: Genome-Wide Identification and Evolutionary Analysis of the SBP-Box Gene Family in Castor Bean
Source: PLoS One. 2014 Jan 22;9(1):e86688. doi: 10.1371/journal.pone.0086688 (PMC3899293; doi:10.1371/journal.pone.0086688)
Supplement: Table S1 — Summary of information on Arabidopsis SPL genes used in this study. (DOC) [file pone.0086688.s004.doc]

**Table S1** Summary of information on *Arabidopsis* SPL genes used in this study.

| **Gene_name** | **Locus_ID** | **Classification ^a^** | **Length ^b^** | **Domain_sequence** |
| --- | --- | --- | --- | --- |
| *AtSPL1* | At2g47070 | Subgroup Ⅱd | 881 | VCQVENCEADLSKVKDYHRRHKVCEMHSKATSATVGGILQRFCQRFCQQCSRFHRFHLLQEFDEGKRSCRRRLAGHNKRRRKTNPE |
| *AtSPL2* | At5g43270 | Subgroup Ⅱa | 396 | HCQVEGCNLDLSSAKDYHRKHRICENHSKFPKVVVSGVERRFCQRFCQQCSRFHRFHCLSEFDEKKRSCRRRLSDHNARRRKPNPG |
| *AtSPL3* | At2g33810 * | Subgroup Ⅱg | 393 | VCQVESCTADMSKAKQYHKRHKVCQFHAKAPHVRISGLHQRFCQRFCQQCSRFHRFHALSEFDEAKRSCRRRLAGHNERRRKSTTD |
| *AtSPL4* | At1g53160 | Subgroup Ⅱg | 927 | LCQVDRCTADMKEAKLYHRRHKVCEVHAKASSVFLSGLNQRFCQRFCQQCSRFHRFHDLQEFDEAKRSCRRRLAGHNERRRKSSGE |
| *AtSPL5* | At3g15270 | Subgroup Ⅱg | 359 | LCQVDRCTVNLTEAKQYYRRHRVCEVHAKASAATVAGVRQRFCQRFCQQCSRFHRFHELPEFDEAKRSCRRRLAGHNERRRKISGD |
| *AtSPL6* | At1g69170* | Subgroup Ⅱc | 1035 | LCQVYGCSKDLSSSKDYHKRHRVCEAHSKTSVVIVNGLEQRFCQRFCQQCSRFHRFHFLSEFDDGKRSCRRRLAGHNERRRKPAFY |
| *AtSPL7* | At5g18830 * | Group Ⅰ | 354 | RCQVPDCEADISELKGYHKRHRVCLRCATASFVVLDGENKRYCQRYCQQCGKFHKFHLLPDFDEGKRSCRRKLERHNNRRKRKPVD |
| *AtSPL8* | At1g02065 * | Subgroup Ⅱe | 988 | RCQAEGCNADLSHAKHYHRRHKVCEFHSKASTVVAAGLSQRFCQRFCQQCSRFHRFHLLSEFDNGKRSCRKRLADHNRRRRKCHQS |
| *AtSPL9* | At2g42200 * | Subgroup Ⅱb | 359 | RCQVEGCGMDLTNAKGYYSRHRVCGVHSKTPKVTVAGIEQRFCQRFCQQCSRFHRFHQLPEFDLEKRSCRRRLAGHNERRRKPQPA |
| *AtSPL10* | At1g27370 | Subgroup Ⅱa | 419 | RCQIDGCELDLSSSKDYHRKHRVCETHSKCPKVVVSGLERRFCQRFCQQCSRFHRFHAVSEFDEKKRSCRKRLSHHNARRRKPQGV |
| *AtSPL11* | At1g27360 * | Subgroup Ⅱa | 131 | RCQIDGCELDLSSAKGYHRKHKVCEKHSKCPKVSVSGLERRFCQRFCQQCSRFHRFHAVSEFDEKKRSCRKRLSHHNARRRKPQGV |
| *AtSPL12* | At3g60030 | Subgroup Ⅱd | 174 | CCQVDNCGADLSKVKDYHRRHKVCEIHSKATTALVGGIMQRFCQRFCQQCSRFHRFHVLEEFDEGKRSCRRRLAGHNKRRRKANPD |
| *AtSPL13* | At5g50570 * | Subgroup Ⅱf | 181 | ICLVDGCDSDFSNCREYHKRHKVCDVHSKTPVVTINGHKQRFCQRFCQQCSRFHRFHALEEFDEGKRSCRKRLDGHNRRRRKPQPE |
| *AtSPL14* | At1g20980 * | Subgroup Ⅱd | 405 | MCQVDNCTEDLSHAKDYHRRHKVCEVHSKATKALVGKQMQRFCQRFCQQCSRFHRFHLLSEFDEGKRSCRRRLAGHNRRRRKTTQP |
| *AtSPL15* | At3g57920 | Subgroup Ⅱb | 801 | RCQVEGCRMDLSNVKAYYSRHKVCCIHSKSSKVIVSGLHQRFCQRFCQQCSRFHRFHQLSEFDLEKRSCRRRLACHNERRRKPQPT |
| *AtSPL16* | At1G76580 | Subgroup Ⅱd | 333 | KCQVDNCKEDLSIAKDYHRRHKVCEVHSKATKALVGKQMQRFCQQCSRFHLLSEFDEGKRSCRRRLDGHNRRRRKTQP |
| *AtSPL17* | At5g50670 | Subgroup Ⅱf | 375 | ICLVDGCDSDFSNCREYHKRHKVCDVHSKTPVVTINGHKQRFCQRFCQQCSRFHRFHALEEFDEGKRSCRKRLDGHNRRRRKPQPE |

^a^ the classification is described as our previous study [1].

^b^ the length indicates the protein length of each gene.

Note: The genes marked by the asterisk in the table were the respective members used for the query in this study.

1. Ling LZ, Zhang SD (2012) Unraveling the distribution and evolution of *miR156*-targeted SPLs in plants by phylogenetic analysis. Plant Diversity and Resources 34: 33-46.
